# Supplementary material for: Bioinformatic Multi-Strategy Profiling of Congenital Heart Defects for Molecular Mechanism Recognition
Source: Int J Mol Sci. 2024 Nov 9;25(22):12052. doi: 10.3390/ijms252212052 (PMC11594028; doi:10.3390/ijms252212052)
Supplement: Supplementary file 1 [file ijms-25-12052-s001.zip › Oliveira et al., 2024 - Supplemental Tables and Figure legends.pdf]

Table S1. Complete list of genes identified via Gene Ontology;

Table S2. Complete list of genes identified via Human Phenotype Ontology;

Table S3. Details of the datasets included in the study;

Table S4. Complete list of genes differentially expressed;

Figure S1: Network comprising experimental evidence of protein-protein interactions in the Gene Ontology (GO) and Human Phenotype Ontology (HPO), considering genes in common between the GO and HPO repositories. Red colors indicate genes identified HPO, purple colors indicate genes identified GO, and blue nodes indicate genes in common in the repositories.
