# Supplementary material for: Bioinformatic Multi-Strategy Profiling of Congenital Heart Defects for Molecular Mechanism Recognition
Source: Int J Mol Sci. 2024 Nov 9;25(22):12052. doi: 10.3390/ijms252212052 (PMC11594028; doi:10.3390/ijms252212052)
Supplement: Supplementary file 1 [file ijms-25-12052-s001.zip › Oliveira et al.,2024_Figure S2.pdf]

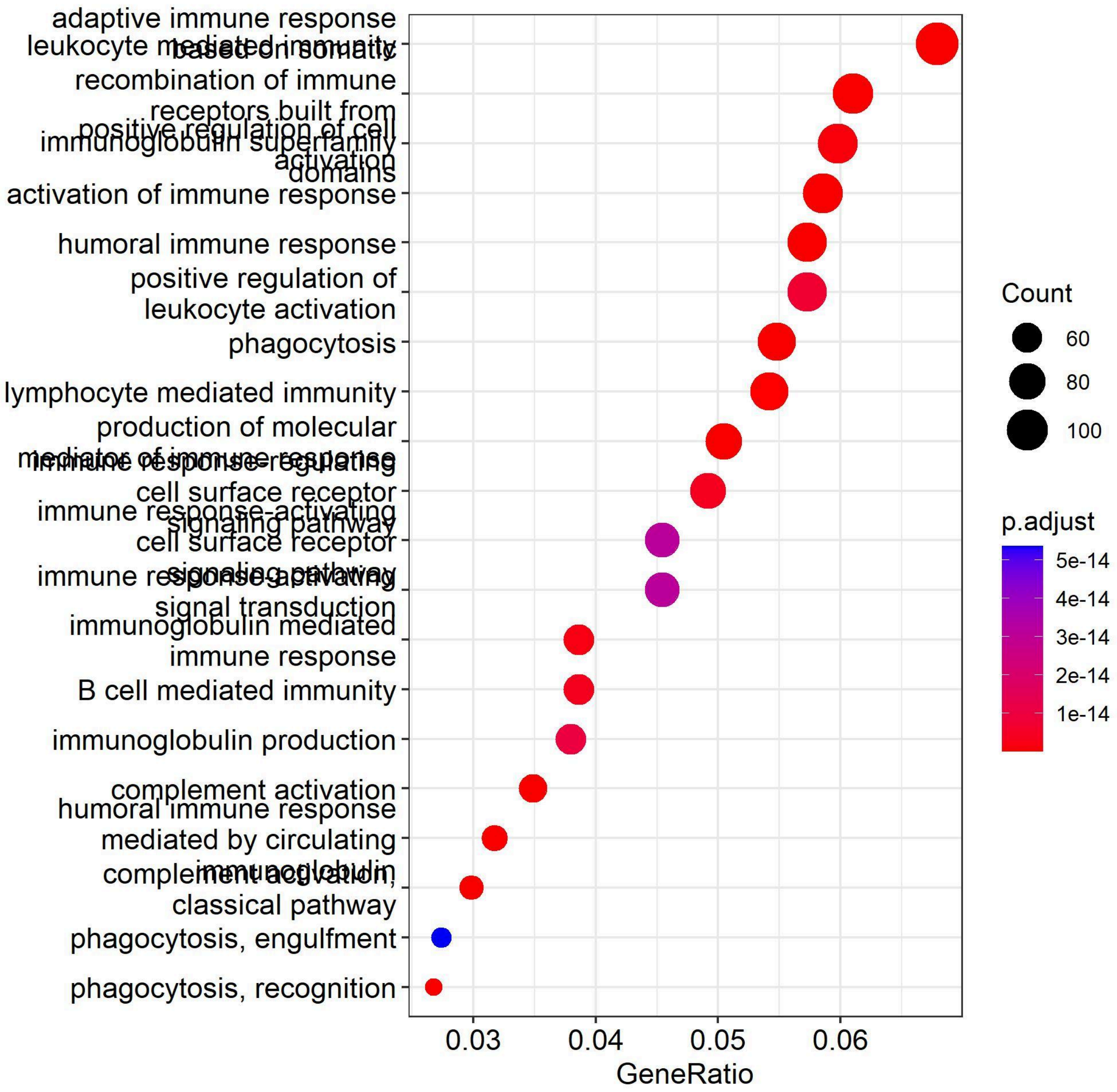

Figure S2a: The ontologies identified for the Tetralogy of Fallot studies.

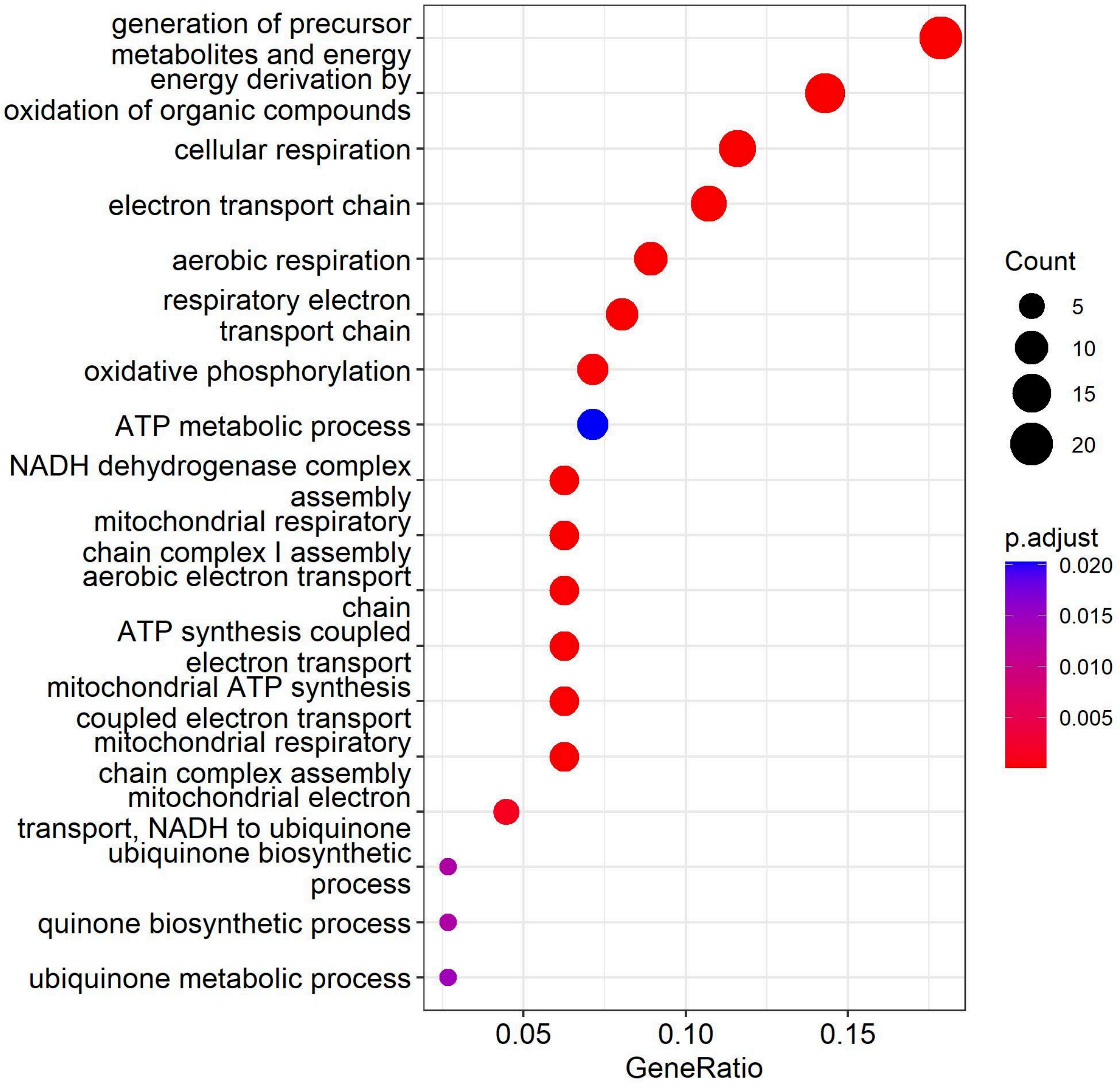

Figure S2b: The ontologies identified for the Hypoplastic Left Heart Syndrome studies.

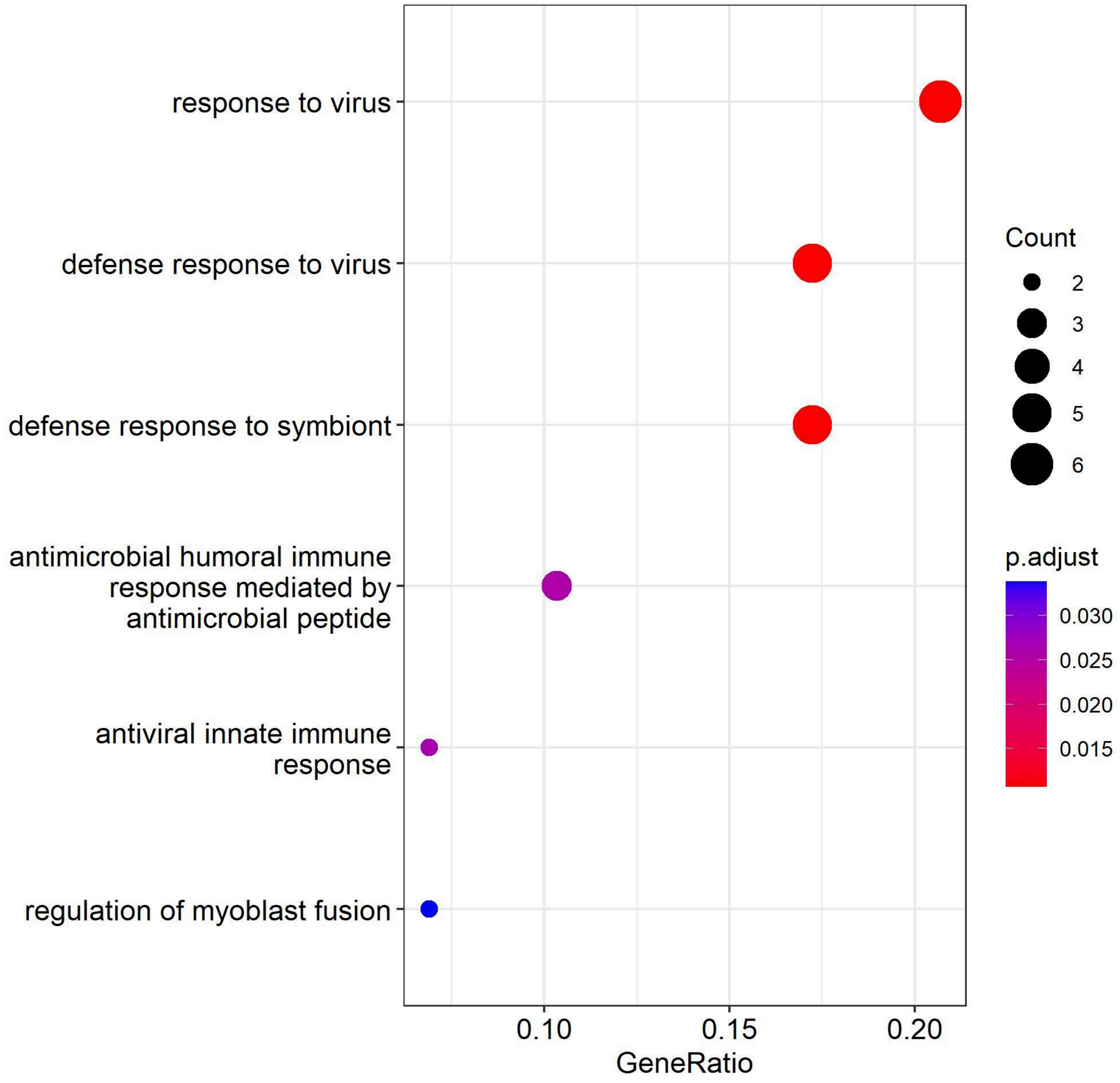

Figure S2c: The ontologies identified for the Trisomy 21/CHD studies.
