# Supplementary figures and images for: Bioinformatic Multi-Strategy Profiling of Congenital Heart Defects for Molecular Mechanism Recognition
Source: Int J Mol Sci. 2024 Nov 9;25(22):12052. doi: 10.3390/ijms252212052 (PMC11594028; doi:10.3390/ijms252212052)

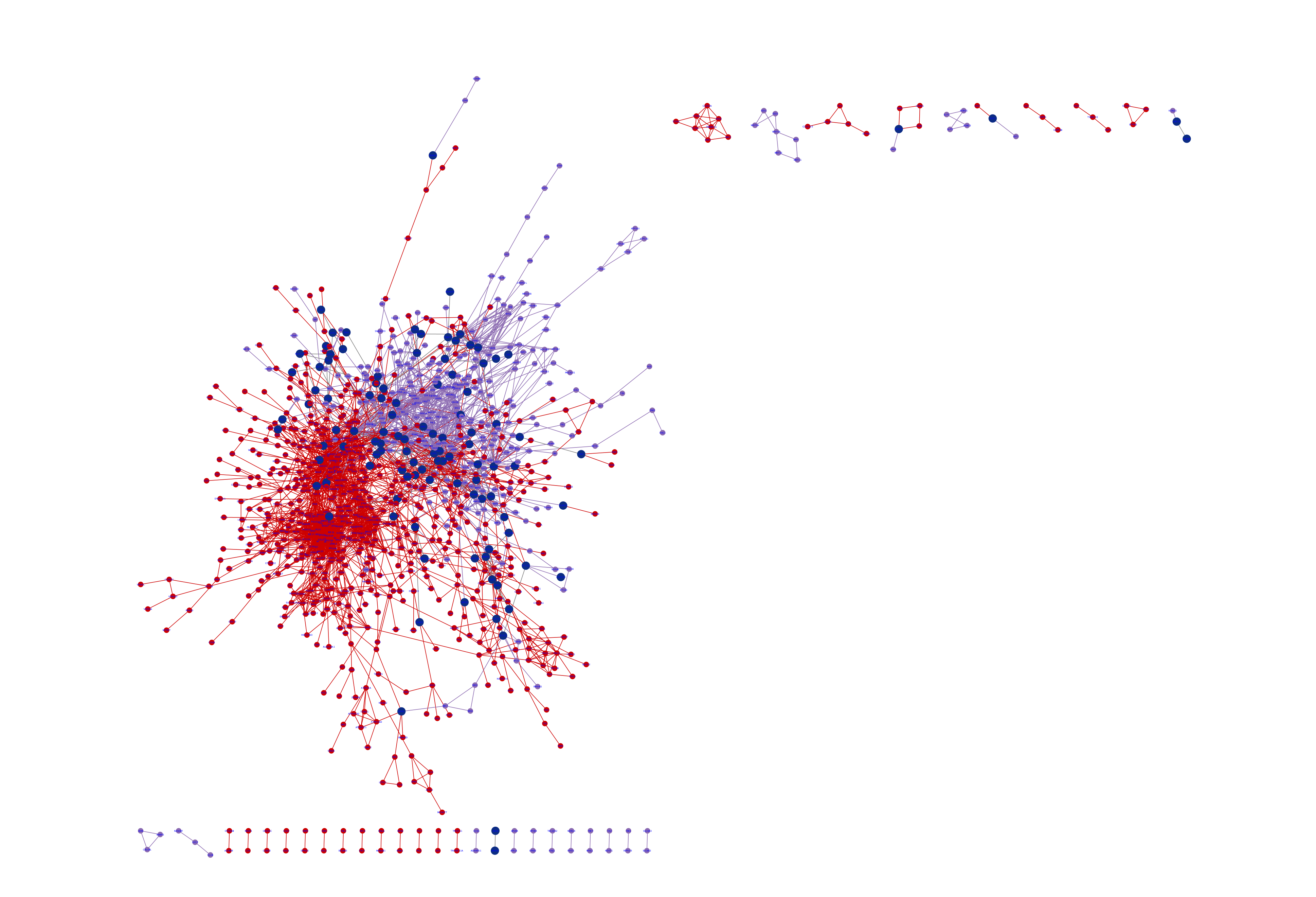

Supplement: Supplementary file 1 [file ijms-25-12052-s001.zip › Oliveira et al., 2024_Figure S1.png]
